# Exploring Sacred Moments in Hospitalized Patients: An Exploratory Qualitative Study

**DOI:** 10.1007/s11606-022-07999-z

**Published:** 2023-01-17

**Authors:** Martha Quinn, Karen E. Fowler, Molly Harrod, Rachel Ehrlinger, Jason M. Engle, Nathan Houchens, Sanjay Saint

**Affiliations:** 1 School of Public Health, University of Michigan, Ann Arbor, MI 48109-2029 USA; 2 Patient Safety Enhancement Program, University of Michigan and Veterans Affairs (VA) Ann Arbor Healthcare System, Ann Arbor, MI USA; 3 VA Ann Arbor Healthcare System, Ann Arbor, MI USA; 4 Department of Internal Medicine, University of Michigan Medical School, Ann Arbor, MI USA

**Keywords:** sacred moment(s), whole health, spirituality, religiosity in medicine

## Abstract

**Background:**

“Sacred moments” are brief periods of time in which people experience a deep interconnectedness that may possess spiritual qualities and emotions. This concept has been shown to have a positive impact on individuals’ overall well-being and stress in mental health settings. The concept of sacred moments has not been studied in acute care hospital settings.

**Objective:**

To better understand the occurrence of sacred moments among hospitalized patients and their healthcare workers.

**Design:**

An exploratory qualitative study that included in-depth interviews with patients and healthcare workers at two academic medical centers in the Midwestern United States.

**Participants:**

Hospital healthcare workers (e.g., physicians, nurses, ancillary staff) and discharged patients with a recent hospital stay.

**Approach:**

Semi-structured telephone interviews were conducted with 30 participants between August 2020 and April 2021. Interviews were recorded and transcribed before conducting thematic analysis.

**Key Results:**

Both healthcare workers and patients reported having experienced at least one sacred moment. Interview findings were organized into three main domains including (1) several common elements described by participants as marking these moments; (2) benefits experienced by both patients and healthcare workers; and (3) suggestions for fostering sacred moments within the hospital setting.

**Conclusions:**

Among our participants, sacred moments were extremely common with the vast majority reporting to have experienced at least one in their lifetime. These moments were described as profound and important and shared many common elements. Our findings can be used to help recognize, understand, and promote sacred moments between hospitalized patients and healthcare workers.

**Supplementary Information:**

The online version contains supplementary material available at 10.1007/s11606-022-07999-z.

## INTRODUCTION

“Sacred moments” have been described as brief periods of time in which people experience spiritual qualities of transcendence, boundlessness, deep interconnectedness, and spiritual emotions.^1,2^ Others have labeled these moments as “sudden intimacies” with total strangers that can occur at times of crisis or grief and that connect people in both unexpected and powerful ways.^3^ These moments, highly memorable for all parties involved, are described as if time stood still and leave participants with a sense of joy, peace, and empathy for the other person. Individuals who have experienced sacred moments during psychotherapy indicated that the moments had sacred qualities such as “I felt a deep sense of connection with my client” and “All distractions seemed to melt away.”^4^ Research has also shown that these moments, when experienced during psychotherapy, can have positive effects on individuals’ overall well-being and stress.^5,6^

Recently, there has been a growing movement toward non-traditional, patient-centered, holistic approaches to healthcare. The Institute of Medicine’s 2001 landmark report *Crossing the Quality Chasm*^7^ identified patient-centered approaches as one of six essential elements in providing high-quality care. Since then, patient-centered approaches have been widely embraced by health systems across the USA. For instance, the Veterans Health Administration, the nation’s largest health system, recently implemented a “Whole Health” approach^8^ to care that includes personalized, proactive, and patient-driven care focused on the social, emotional, relational, and spiritual needs of patients. The benefits of these approaches have been well documented and include increased patient satisfaction with care, improved health outcomes, and boosts in clinician morale.^9,10^ In 2019, the authors undertook a study to explore the implementation of whole health programs at two affiliated hospitals. The focus of this paper is to describe our findings related to the occurrence of sacred moments, a potential component of a larger whole health program, within the acute care hospital setting.

As hospitals continue to adopt patient-centered approaches, there are gaps in what is known about the factors that encourage and drive these changes.^11^ Similarly, while well documented in the field of psychology,^4,12,13^ little has been published about the occurrence of sacred moments in the broader healthcare field. Our findings help to fill this gap in knowledge and to better understand sacred moment experiences and their effects on patients and healthcare workers.

## METHODS

We conducted an exploratory qualitative study that included in-depth interviews with healthcare workers and patients from two affiliated Midwestern hospitals — a large university hospital and a smaller academically affiliated Veterans Affairs (VA) medical center. The interviews were part of a larger study exploring the possible implementation of various whole health services into the care of hospitalized patients. While the larger study explored all aspects of whole health, data used for this manuscript originated from responses to a subset of interview questions that focused on the spiritual needs and perceptions of patients and healthcare workers and specifically their experiences with, and views of, sacred moments. Appropriate Institutional Review Board approval or exemption status was obtained from each hospital.

### Participants and Data Collection

We used purposeful sampling to identify key stakeholders, ensuring that healthcare workers in various roles (e.g., physicians, nurses, social workers, chaplains) were represented. Potential subjects were identified based on work role and invited to participate through email. If they agreed, a telephone interview was scheduled at their convenience.

Patients were identified using a convenience sample, as patient recruitment varied between the two hospitals. At the VA, hospital medicine physicians were asked to recommend a few recently discharged patients (primarily within the previous 1 to 3 months), who were alert and oriented, and were thought to be good interview candidates. Four potential subjects were identified by recommendation. The majority of patients were identified from a list of recent discharges from the hospital electronic medical record system. Patients with terminal diagnoses or who were incapable of providing informed consent were excluded. At the university hospital, patients were recruited through the Office of Patient Experience. Potential subjects were mailed a letter explaining the study and inviting them to participate. This was followed by a telephone call by study staff to answer questions and determine participant interest. If there was no response after three call attempts, it was considered a passive decline. Interviewees were offered a $25 gift card for their participation. Remuneration was not allowed for VA staff participating during working hours, per Federal statute.

A total of 24 healthcare workers and 22 patients were invited to participate. Of these, 1 healthcare worker and 11 patients either declined or were non-responsive. Additionally, 4 individuals who completed interviews were omitted from this analysis because they were not asked the subset of questions related to sacred moments due to time constraints or other reasons. A semi-structured interview guide was used, and questions specific to sacred moments are included in Appendix Table 1. Individual telephone interviews were conducted between August 2020 and April 2021 by a trained interviewer (MQ) and the study coordinator (KEF). Interviews lasted on average 43.1 min (range, 20.5 to 61.3 min). Interviews were audio recorded, transcribed, and verified for accuracy.

### Data Analysis

We performed thematic analysis on textual data from interview transcripts.^14^ This approach to qualitative analysis has been widely used in health research,^15^ and is useful in gaining an in-depth understanding of complex phenomena and differing viewpoints and experiences.^14,16^ We began by reading and re-reading interview transcripts to become familiar with the data, note initial ideas, and ensure content immersion. Two members of the team with extensive qualitative methods experience (MQ, MH) independently reviewed one transcript to begin preliminary codebook development. Codes were inductively developed based on the content of the text and systematically cataloged in the codebook, along with verbatim quotations. The two then met to discuss the coded transcript and refine the codebook. Differences in applied coding were discussed, and agreement was reached by consensus. This process was repeated for 2 more transcripts. Any newly added codes were applied to previously reviewed transcripts. This process increased inter-coder reliability and ensured consistent coding across the dataset. The remainder of the transcripts were then divided between the primary coders for initial coding. A third study team member (RE) conducted a secondary review of each coded transcript. Any discrepancies were discussed and resolved through agreement until all transcripts were completed. NVivo 12 software (QSR International Pty Ltd, 2018) was used for data management and to develop code summary reports. These code reports, which included all data aggregated under the same code, were then analyzed by the study team to identify patterns in the data. Findings were organized into main domains of exploration (e.g., common elements of sacred moments, benefits to patients and healthcare workers, suggestions for fostering moments) which were derived from the study aims and tracked closely to the interview guide.

## RESULTS

In total, we interviewed 30 individuals about their sacred moment experiences, including 8 patients and 22 healthcare workers (9 physicians, 6 nurses, and 7 other staff such as social workers or chaplains). Sixty percent (*n*=18) of participants were female, and the majority (*n*=24) were non-Hispanic/White. Two-thirds were recruited from the VA medical center and one-third were from the university hospital.

Through these interviews, we found that sacred moment experiences were common among healthcare workers and patients, with the vast majority of participants reporting that they had experienced at least one of these moments within the hospital setting at some point within their lifetime. While some participants had not heard of the specific term “sacred moment,” they immediately connected with the concept when it was explained and went on to describe their own experiences. Interview findings were organized into three main domains: (1) common elements described by participants as marking these experiences; (2) benefits to both healthcare workers and patients; and (3) suggestions for fostering sacred moments. A table listing the findings under each of these domains, along with additional quotations, can be found in Appendix Table 2.

### Domain 1: Common Elements of Sacred Moments

This domain describes the common elements of the sacred moment experiences reported. While descriptions varied among participants, interviewees talked about several common elements that marked these moments including the following: an interconnectedness between healthcare workers and patients; intense emotions and empathy; a sense of awe and spirituality; occurrence during death or near-death experiences or during open and relaxed moments; and profound meaning.

#### Interconnectedness between Healthcare Worker and Patient

It’s kinda like the Maya Angelou quote, ‘I don’t remember what was said, but I remember how it felt.’ (nurse)

Healthcare workers and patients talked about establishing a bond, having a natural rapport, and connecting on a personal level with each other during sacred moments. They were described as times in which they were not bound by their role as a patient or healthcare worker. There was a leveling or equalizing between individuals and a deep human connection. In particular, patients discussed feeling valued as a “whole person” during these experiences, rather than just someone with “cancer” or another health condition. In some cases, this involved sharing similar interests or hobbies or talking about family. One healthcare worker noted that this human connection was very important during the COVID-19 pandemic during which hospital staff became “surrogate family” to patients.

#### Intense Emotions and Empathy

They create empathy in ways that other interactions don’t. (physician)

Both healthcare workers and patients described these moments as “intense” and “emotional,” with some participants becoming tearful during interviews when retelling their experiences. A patient recounted an experience in which her doctor provided emotional support by being present and holding her hand when she came out of surgery.

#### Sense of Awe and Spirituality

It was a very spiritual moment… it was like being in the Garden of Gethsemane. (nurse)

Healthcare workers and patients described these moments as “transcending,” “spiritual,” “moving,” and with a sense of “awe.” Though some found it difficult to describe, they reported experiencing something greater than their physical circumstances, something profoundly spiritual. Some patients mentioned times in which their clinicians prayed with them and that this act was very meaningful to them.

#### During Death or Near-Death Experiences

Every now and then you’re dealing with what is the meaning of life. (physician)

Healthcare workers reported that these experiences of sacred moments occur more commonly during end-of-life care. One palliative care physician said, “I experience it with almost every patient.” Participants pointed out that dying patients may be reflecting on their life journeys and their mortality, leaving them more open to deep connections and meaningful moments. Further, healthcare workers caring for terminal patients said that sometimes when there are no further medical treatment options, they emphasize other tools such as listening, providing emotional support, and spending more time with patients. While mostly reported by healthcare workers, one patient described a sacred moment during a near-death experience as comforting and said, “I knew that I was afraid but yet I wasn’t.”

#### Open, Relaxed Moments

I just sat there and listened. (hospital staff)

Healthcare workers and patients reported that these experiences occurred during serene and calm moments when both parties were “open” and “present.” They described sacred moments as pauses in time that were not filled with physical examinations or structured medical discussions, but rather shared, unstructured time spent together. Healthcare workers reported the importance of really listening to patients and understanding their needs and concerns during this time. Patients said they wanted healthcare workers to try and understand their concerns and to see the situation from a patient’s perspective. An example included when a nurse remained, after the physician team left, to try and understand why an elderly patient was declining chemotherapy. The nurse said, “I just stayed and listened, and finally he said, ‘I’ve already done that, I’m not doing it again.’”

#### Profoundly Meaningful

I can’t even describe … how significant it was. (nurse)

These experiences were described as important and deeply meaningful. For patients, “profoundly meaningful” meant that they truly felt their healthcare provider cared for them. One patient recounted a story about being nervous to be in the hospital and how meaningful it was when his doctor spent time with him. For healthcare workers, these moments reminded them of why they went into healthcare and made them feel like they were fulfilling their calling.

### Domain 2: Benefits of Sacred Moments

According to our interviewees, sacred moment experiences had deep positive impacts on both patients and healthcare workers including reducing patient anxiety, improving care satisfaction/job satisfaction, buffering against clinician burnout, and strengthening patient-provider bonds.

#### Patient Benefits

The only way I could explain that is it was easier to breathe… (patient)

Patients reported that these experiences helped to reduce their anxiety and fear, particularly at a time when their stress was high due to being hospitalized and having serious health concerns. Other patients said that receiving emotional support from their care team during difficult times provided comfort. Examples included both verbal reassurance and empathy, as well as nonverbal support such as touching their arm and making eye contact. A few patients talked about how these connecting moments reduced their stress, instilled hope, and brought them peace.

That was something because he [the doctor] totally relaxed me because I was a nervous wreck being in the hospital you know, and he just took the time to care … and took the time to show that he cared and that’s what got me through it… (patient)

One physician reported that these moments also increase patients’ satisfaction with their care and care team.

… I think it’s just amazing how it sort of shifts their view of their healthcare team and their view of kind of, I don’t know, I think sometimes their satisfaction with what’s going on in their healthcare. (physician)

#### Healthcare Worker Benefits

The reason I went into medicine. (physician)

During interviews, healthcare workers said these moments provided validation, affirmed their decision to go into medicine, and gave them energy to go on during difficult times. Interviewees talked about clinician fatigue and burnout and how these experiences can help buffer against the grind of medicine.

Well, I mean it gives you the energy and I guess it makes you feel like what you are doing is right and that your job is more than a job at that time. (nurse)

It was also reported that taking a moment with a patient after they receive serious news or with a family member after a patient’s death helps everyone emotionally process difficult and intense experiences. Both healthcare workers and patients reported that sacred moments strengthened the bonds and trust between them. Several patients talked about visiting their favorite nurse when they returned to the hospital for subsequent visits. Healthcare workers reported visiting patients as they moved to other units in the hospital or returning to a patient’s room later in the day to talk one-on-one.

I remember sitting by his bed and putting my hand on his hand and him putting his other hand on top of my hand and just making … eye contact and having a moment where I just really valued him as a person and as a patient, and I felt that he very much valued me as his nurse, and there was a lot of just trust and connection in that moment. (nurse)

### Domain 3: Suggestions for Fostering Sacred Moments

While participants were optimistic about increasing opportunities for sacred moments, they also identified several challenges. They recognized that healthcare workers are extremely busy and have little time to spend with patients beyond providing critical and timely medical care. Additionally, staffing shortages, long hours, and heavy patient caseloads were mentioned. Clinicians pointed out that acute care settings are also accompanied by constant transitions in care teams and shorter patient stays, all of which lead to difficulty in establishing relationships. Healthcare workers also noted that the hospital environment, in general, does not promote a sense of transcendence, spirituality, or deep connection. Interviewees pointed out that healthcare workers may be reluctant to have these conversations because they feel ill-equipped and untrained to pray with patients or offer emotional support. One physician mentioned that the structure of morning rounds, the primary time clinicians see their patients, is not conducive to sacred moments because they are rushed, focused primarily on pressing medical needs, and typically include large rounding teams. Despite these challenges, interviewees had several suggestions for fostering these moments which included both system-level changes and individual, clinician-led modifications.

#### Suggestions for System-Level Changes

It’s really a healthcare systems issue. (physician)

While acknowledging recent staffing shortages and financial constraints, healthcare workers reported that recruiting and hiring more staff would solve many problems. A few healthcare workers also pointed out that smaller hospitals often have lighter patient caseloads, which allows clinicians to spend more time with patients.

…having an average patient load of 6-8 is low for a hospitalist, but it does allow me to really invest time in patients…I’m able to have those personal relationships and really know who my patients are and what matters to them. (physician)

Clinicians suggested developing mentoring and educational opportunities for healthcare workers to become familiar with sacred moments, to recognize how to foster them with patients, and to become more comfortable having emotional and spiritual connections with patients. Two mentoring programs were recognized as being very valuable to physicians, and these included resident physicians engaging in a palliative care rotation, and medical students shadowing a chaplain during an intensive care unit rotation. These programs allow trainees to observe sacred moments and how experienced clinicians navigate them. Others suggested including sacred moments in medical and nursing school curricula.

Some clinicians stressed the need to encourage healthcare workers to talk with each other about their experiences — discussion that does not often happen, according to interviewees. Clinicians said these discussions could help them process difficult patient care experiences and support each other.

Interviewees also discussed the need for a cultural shift in inpatient care from a traditional “find-it, fix-it” approach which focuses on treating individual, isolated medical problems to one that is more holistic, connected, and patient-driven. This shift includes recognizing and addressing not just the physical needs of a person, but also their emotional, social, spiritual, and mental health needs.

#### Individual, Healthcare Worker-Led Modifications

… just being able to slow down and show up to each moment. (hospital staff)

In addition to system-level changes, individual, clinician-led modifications were also suggested to help nurture sacred moments and included slowing down, giving emotional support, making eye contact, and exploring ways to make a human connection with patients.

You know, there are ways you can give a patient the impression that you have all the time in the world even though you really don’t. You know you can just sit down for a minute or make eye contact or stand closer or something. I think all the doctors and the residents really need to work on that, come close, yeah, don’t stand at the end of the bed. (patient)

Healthcare workers offered some examples of how they carved out time during patient care to sit briefly with a patient and listen or pause and let the patient ask questions. Some physicians discussed their practice of returning to a patient’s room later in the day, after the hectic morning rounds, to talk with patients and answer their questions.

You don’t spend your day running from cardiac arrest to cardiac arrest, and so if a patient wants to spend 10 minutes with you, you can, you can give them 10 minutes. (physician)

## DISCUSSION

Our study found that sacred moments were extremely common, with the vast majority of healthcare workers and patients reporting that they had experienced at least one in their lifetime. Our three main findings included the following: (1) there were several common elements that comprised and defined these experiences; (2) patients and healthcare workers experienced several benefits from these moments; and (3) participants had suggestions for fostering these moments in the busy hospital setting.

Our findings are consistent with the work of Pargament and colleagues, despite the fact that they focused on sacred moments during psychotherapeutic interactions.^1^ Specifically, the common elements of sacred moments reported in our study — such as experiencing transcendence, interconnectedness, and strong emotions — were similar to those experienced during psychotherapeutic interactions.^4^ Further, the benefits to patients and healthcare workers reported in our study are consistent with prior research noting therapeutic gains, such as improved relations between patient and clinician, increased patient satisfaction, and bolstered mental health status.^4^ Additionally, Radetsky wrote about “sudden intimacies” decades ago — a concept similar to sacred moments in that it recognizes the intensity of these moments, the clarity they can provide, and the emotional rewards of connecting deeply with another.^3^ The unique contribution of our research is the exploration of these connecting, meaningful, and sometimes spiritual moments within the hospital setting.

Central to this discussion are the overall concepts of spirituality and religion^17^ and whether they belong in healthcare systems. While considered a taboo topic by some, spirituality has been reported to be important to both hospitalized patients and healthcare providers.^18,19^ A recent study showed that 51% of physicians report themselves as “religious” and 25% as “spiritual.”^20^ Almost one-third cite religious or spiritual beliefs as an influence in their decision to become a physician.^20^ Additionally, most patients feel that physicians should be aware of their patients’ religious and spiritual beliefs.^21^ Despite these statistics, our interviews revealed providers’ reluctance to pray with or discuss spiritual matters with patients. Some providers felt unprepared or untrained to pray with patients. Others indicated a fear of accidentally offending them. To alleviate healthcare workers’ concerns and provide the care patients desire, mentoring and training programs designed to enhance healthcare workers’ skills and comfort in discussing religion and spirituality are needed.^22^

This study should be interpreted within the context of the following limitations. First, our sample size was small and included only 30 individuals, only 8 of whom were patients, located at two hospitals in one geographic location, and therefore, findings may not be generalizable. Second, two of the patients were recommended as potential participants by their physicians, which could introduce selection bias. Third, our interviews took place during the COVID-19 pandemic, which was stressful for both patients and healthcare workers and may have affected some of the participants’ views.

Findings from this study are both timely and relevant. The hospital setting can be a chaotic, stress-inducing environment for both patients and healthcare workers, which has only been amplified by the COVID-19 pandemic. During COVID-19 surges, due to visitation restrictions, patients were often alone in their rooms having to ask questions, advocate for themselves, and make decisions without the immediate support of family and friends. Healthcare workers often dealt with staffing shortages, juggled large and often complex patient caseloads, and experienced high levels of burnout^23,24^ and depersonalization symptoms.^25,26^ While exploratory in nature, this work has relevance to some of the defining problems in healthcare today including clinician burnout and patient satisfaction. Both patients and their care teams are under stress. Veterans, for example, continue to experience high rates of depression, post-traumatic stress disorder, and suicide.^27^ Healthcare workers increasingly report high levels of stress and burnout and lower levels of job satisfaction.^28^ New and innovative approaches are needed now to address these comorbid concerns, and our findings can be used to inform future studies in this area. Specifically, it will be important to assess the generalizability of our results and whether sacred moments are as common in other healthcare facilities. Further, it would be interesting to explore how sacred moments may differ in frequency and experience within different types of healthcare workers. Additional studies are also needed to explore interventions to cultivate sacred moments in the healthcare setting. As suggested by our interviewees, interventions could focus on educating clinical staff about sacred moments, encouraging discussion of these moments with coworkers to increase awareness, and activities to improve patient-provider relationships to foster these moments. Our findings indicate that sacred moments have the potential to improve patient satisfaction and well-being while reducing healthcare worker burnout.

## Supplementary Information

ESM 1 (DOCX 23.7 kb)

## Data Availability

The analysis during the current study is available from the corresponding author on reasonable request.
