# Supplementary material for: Exploring Sacred Moments in Hospitalized Patients: An Exploratory Qualitative Study
Source: J Gen Intern Med. 2023 Jan 17;38(9):2038–44. doi: 10.1007/s11606-022-07999-z (PMC9845021; doi:10.1007/s11606-022-07999-z)
Supplement: Supplementary file 1 — (DOCX 23.7 kb) [file 11606_2022_7999_MOESM1_ESM.docx]

**APPENDIX TABLE 1**

**Key Concepts and Questions About Sacred Moments from the Study Interview Guide**

| 1. Have you heard of the term Sacred Moments? What does it mean to you? ***** |
| --- |
| 1. Have you personally ever experienced a sacred moment? |
| 1. [If yes] Please take a moment to describe that for me. What was that like for you? What made it sacred? |
| 1. How could you cultivate or increase the chances of experiencing these sacred moments with your (patients/healthcare providers)? |
| 1. How do patients/providers benefit from these moments? |

* If interviewee responded as “no” to the first question, the interviewer provided the following description: These are moments that people have described as sacred, memorable, connecting moments that patients and providers share together. Sometimes these can happen spontaneously during times of crisis or great sadness or conversely during times of great joy.

**APPENDIX TABLE 2**

**Sacred Moments Domains, Findings, and Exemplary Quotes**

| 1. **Common Elements of Sacred Moments** | |
| --- | --- |
| - Interconnectedness between healthcare worker and patient | - *“So, you’re talking about these very special moments, where you kind of go off-the-record with a patient, where you bond as two human beings in a shared moment where you, as a clinician… allow yourself to have kind of that vulnerability.” (Physician)* - *“It was really a surprise and doctor [name redacted] sat down with me for like 20 minutes… and that was like a great connecting moment.” (Patient)* - *“So the one that stands out to me, she ended up dying but not that day but later, but we had these, all kinds of great conversations about her kids, and she was writing letters to her young girls so when they graduated high school they would be able to read them from their mom, and it was just like this, like heart-wrenching experience but it was nice to connect with someone as a human as opposed to labeling that person as just being in pain, you know or having cancer, and those things sustain you.” (Physician)* |
| - Intense emotions and empathy | - *“We just had this moment where it was like… I can’t fix it, I can’t make the cancer go away. I’m as human as you are. This could be happening to me or my mom, or… my sister.”* *(Physician)* |
| - Sense of awe and spirituality | - *“It was a very spiritual moment… it was like being in the Garden of Gethsemane… and I was just like, ‘Okay, I’m just going to go with this,’ but you know it’s like you walked out of there and it was like your mind was blown, that’s the only way I can describe it.” (Nurse)* - *“I think spirituality is an important component of life and I think that others probably think that too, and so I would think that, you know, continuing to offer that type of service [opportunities to pray or visits from chaplains] is important.” (Patient)* - *“… physicians would say they don’t have the expertise … but around half of the patients in the hospital… would like to have their physician pray with them. That’s a stunning statistic to me.” (Physician)* |
| - During death or near-death experiences | - *“There have been times when I can do nothing else medically for my patients, they have a terminal disease, or they have reached the limits of medical science; and there’s nothing else we can do to treat them besides help them to become comfortable. And sometimes for those patients I’ve asked about their interest in music and what they like and literally taken them for a piano concert in the hospital … whether by themselves or had their family members come…* *I felt that I was doing good for them. Hearing these people kind of just break down, tears of kind of beauty of the moment, tears of being able to spend this time with their family. I think it was very special to them that a physician would be willing just to do that and try to give to them when they have nothing else to give.” (Physician)* - *“She ended up being told by the doctor that they couldn’t save her, and she had the decision [of] when they were going to stop the drips and eventually, she was going to fall asleep and not wake up and she was going to die. And she asked me, ‘will you hold my hand if I die?’ And so yes, that’s what I did. It was during a time when there were no visitors, so she didn’t want to die alone … it kind of hit me more so after, but like during the time I had a lot of peace about it.”* *(Staff)* |
| - Open, relaxed moments | - *“Just in three words - listen, listen, listen. Now providers by nature, what they do, they have to teach, they have to instruct, they have to direct, they have to get folks to do what needs to be done for their physical bodies but the bedside manner of the attending and listening will go very far as it relates to building those relationships…” (Staff)* |
| - Profoundly meaningful | - *“It was just so awesome to be able to be there with him and experience that. I would definitely call that a spiritual moment or a sacred moment. I remember going home and telling my husband, ‘you know, this is why I am doing what I am doing. This is what it’s all about.’”* *(Nurse)* |
| 1. **Benefits of Sacred Moments** |  |
| - Benefits to patients:   - Reduces anxiety and fear   - Provides comfort and emotional support, hope   - Strengthens bonds with care team   - Improves satisfaction with care | - *“…spirituality is important. For people that don’t have it, believe me while they are laying in that bed with all kinds of things to think about, hopefully it enlightens them to start thinking spiritually and when you start thinking spiritually, it helps mentally and mentally helps physically, everything that we’ve talked about is in a circle.” (Patient)* - *…the care that I received and the concern for my well-being and the steps that they [providers] were taking, it always felt very genuine, that they were truly concerned about me and they wanted to help me… I felt that we were connected.” (Patient)* |
| - Benefits to healthcare workers:   - Provides validation and affirmation   - Buffers against burnout   - Strengthens bonds with patients   - Helps emotionally process life and death experiences | - *“Spirituality isn’t engrained in [medical school] which becomes problematic because this is how they make meaning out of their vocation and their livelihood and when the grind of medicine hits, how do you feel a sense of worth and validation and like you are fulfilling your calling? For a lot of them it’s a calling and how do you maintain that? We should buffer against burnout, but if we don’t teach them how to do that…” (Staff)* - *“I think these discussions are important… we had a patient that passed in CPR, I think that was a moment that was shared with all of us and we took some time to reflect on that and there is always that process after where the family is involved. You know we collectively had that moment.” (Physician)* |
| 1. **Suggestions for Fostering Sacred Moments** | |
| - System-level changes   - Lighter caseloads, increasing staff   - Education and mentoring programs   - Culture shift - Individual, healthcare worker-led modifications   - Slowing down and taking a moment   - Provide emotional support   - Make a human connection | - *“It’s really a healthcare systems issue as opposed to like anything else… if a doctor has to spend 6 of their 12 hours at work doing documentation, it makes it really hard to feel like you have the time to have these moments...” (Physician)* - *“I mean I’ve had residents and medical students cry during those [sacred moment shadowing] interactions… I guarantee that that experience stays with them, and probably role-modeling is a better teaching tool for them than it would be, you know having a lecture.” (Physician)* - *“You know you need someone who is really comfortable with communication, mature, and comfortable with you know, people being emotional, and gosh in despair. You know usually what I tell people is, ‘you know you’re going to be a palliative care doc when you know a family that is sad and instead of running away, you run towards it, right?’ The firefighter analogy but it’s very true. You need to have a certain degree of self-confidence, having done your own kind of soul-searching and emotional maturing in order to really engage in that space.” (Physician)* - *“I had surgery last year … and I woke up and my primary care doctor had come in to visit me. I didn’t even know she was in the room and I felt someone holding my hand and it was my doctor, there to provide, I guess in her own way spiritual or emotional support.” (Patient)* - *I’d say [the providers] exhibited a great deal of caring towards me and I always felt that when they were talking with me, they weren’t just throwing out a boilerplate right, they were truly concerned … they weren’t in some kind of hierarchy or anything.” (Patient)* |
